# EMPATHIC COMMUNICATION WITH OLDER ADULTS DURING DIGNITY THERAPY

**DOI:** 10.1093/geroni/igae098.2311

**Published:** 2024-12-31

**Authors:** Carma Bylund, Mary Kate Koch, Alyssa Crowe, Susan Bluck

**Affiliations:** University of Florida, Gainesville, Florida, United States; University of Florida, Gainesville, Florida, United States; University of Florida College of Medicine, Gainesville, Florida, United States; University of Florida, Gainesville, Florida, United States

## Abstract

Dignity Therapy (DT) is designed to preserve patient dignity through a life story-legacy interview guided by a trained provider. Given the evidence on the important role of empathy in provider-patient interactions, we focused on the provider in dignity therapy interviews with older adults and aimed to assess: 1) relative frequency of use of varying empathic skills; 2) whether use of empathic skills varies by provider and DT modality, and (3) whether older palliative outpatients’ physical functioning status impacted the frequency of providers’ use of empathic communication skills. We analyzed 203 transcribed DT sessions with palliative care cancer outpatients aged 55 years or older, for providers’ use of four empathic skills: acknowledgement, reflection, validation, and empathic self-disclosure. Patients (M=65.78 years; SD=7.43 years) completed the Palliative Performance Scale before the session. Across all providers (n=14), acknowledgement was used most frequently (M =.45, SD =.23), followed closely by validation (M =.43, SD =.24). Use of empathic communication skills did not differ by provider occupation, gender, or DT modality. Multi-level modeling indicated providers used more validation (B = -.12, p <.05) and total empathic communication skills (B = -.19, p <.05) in Dignity Therapy sessions with patients who had lower palliative performance scores (i.e., had reduced physical functioning). Thus, providers seem to adjust their approach to DT interviews based on patients’ physical functioning. More research is needed to understand the impact of empathic communication skills on patient outcomes.

